# Evaluation of a Hydrophobic Coating Agent Based on Cellulose Nanofiber and Alkyl Ketone Dimer

**DOI:** 10.3390/ma16124216

**Published:** 2023-06-07

**Authors:** Nag-Seop Jang, Chi-Hoon Noh, Young-Hwan Kim, Hee-Jun Yang, Hyeon-Gi Lee, HongSeob Oh

**Affiliations:** 1 Department of Civil Engineering, Gyeongsang National University, Jinju 52725, Republic of Korea; bonitosubi@gmail.com (N.-S.J.); tutove@gnu.ac.kr (C.-H.N.); 2 Bricon Lab Inc., Advanced Construction Materials Testing Center, Daegu 42601, Republic of Korea; orrr0213@gmail.com (Y.-H.K.); endorphiny7@gmail.com (H.-J.Y.); hyeongi84@gmail.com (H.-G.L.)

**Keywords:** cellulose nanofiber, hydrophobic, contact angle, concrete durability

## Abstract

In this study, we report on the development and testing of hydrophobic coatings using cellulose fibers. The developed hydrophobic coating agent secured hydrophobic performance over 120°. In addition, a pencil hardness test, rapid chloride ion penetration test, and carbonation test were conducted, and it was confirmed that concrete durability could be improved. We believe that this study will promote the research and development of hydrophobic coatings in the future.

## 1. Introduction

Reinforced concrete structures are the most widely used construction materials in the world due to their high strength and workability at an affordable price [1,2,3].

Water can infiltrate the concrete structure and then erode it. Chloride ions cause severe corrosion to steel rebars in reinforced concrete members, resulting in a significant decrease in mechanical strength and a sharp decline in service life [4,5]. The corrosion of steel rebars causes expansion pressure in the surrounding concrete, causing it to crack, and delamination or detachment of the steel rebars, resulting in a reduction in the cross-section of the steel rebars [6]. Deterioration of the durability of reinforced concrete structures results in significant maintenance costs [7,8,9].

In the United States, annual infrastructure-related costs total USD 22.6 billion, while corrosion costs associated with bridges amount to approximately USD 8.3 billion [10]. In the UK, around 50% of the construction budget is spent on the repair and maintenance of structures, and around 30% of this expenditure is on concrete structures. Traffic delays due to inspection and maintenance programs are estimated to represent between 15 and 40 percent of construction costs [11]. In the Netherlands, the number of bridges requiring maintenance is estimated to increase two- to four-fold over the next 20 years and three- to six-fold over the next 40 years [12].

To improve the durability of buildings and social infrastructure, products such as surface impregnation materials and permeable absorption prevention materials are used on concrete surfaces [13,14,15,16].

When surface-impregnated materials and permeable absorbent materials are used, the environment of use, the type of concrete, economic feasibility, and efficiency are the most important factors in selecting the appropriate products [17]. Therefore, surface-impregnated materials and permeable absorption inhibitors have been developed by utilizing chemical materials such as sodium silicate, epoxy resin, acrylic, and polyurethane. However, if the surface impregnation material and the permeable absorption prevention material lose their function due to long-term deterioration, there are cases where the damaged area needs to be reworked. The deterioration also causes serious environmental pollution by discharging environmental pollutants [14,16,18,19,20].

Research is being actively conducted into eco-friendly repair materials that prevent moisture penetration in marine structures, as well as preventing surface pollution [21,22,23,24].

Cellulose is one of the most common polymeric materials on Earth, and it is renewable and eco-friendly. Cellulose nanocrystal (CNC) is manufactured via chemical treatment, cellulose nanofibril (CNF) is manufactured via mechanical treatment, and bacterial cellulose (BC) is manufactured via biological treatment [25,26,27]. CNF consists of significant amorphous regions, with soft, long chains with widths ranging from ten to a few hundred nanometers, and lengths on the micrometer scale [28,29,30,31]. Furthermore, CNF has low thermal expansion, a high aspect ratio, high-strength characteristics, and good mechanical and optical properties, so it can be used for manufacturing various composite materials [32,33,34]. Therefore, CNF is actively researched in many fields such as medical products, composites, printed electronics, paint, paper, and cosmetics [35,36,37]. However, CNF has poor moisture resistance due to its porous structure and hydrophilic properties, resulting in a sharp decrease in performance under high humidity conditions [38,39].

Various methods for increasing the moisture resistance of CNF are being studied. One method may partially express hydrophobicity by adding alkyl ketene dimer (AKD) to increase the moisture resistance of CNF [40,41,42]. AKD is a substance used in the paper industry and is a crystallized wax with a melting point of 40 to 60 °C depending on the length of the dimer carbon chain [43]. AKD has a structure in which two long alkyl groups are attached to the lactone ring, and hydrophobicity can be imparted to cellulose by an alkyl group [44]. In addition, the sizing mechanism of AKD considers that the lactone ring of AKD reacts with hydroxyl groups of the cellulose surface to generate β-keto esters [45,46]. This creates capillary resistance to liquid penetration into the paper, which possesses macroscopic pores with a radius between 0.1 and 10 μm [47,48,49].

In this study, coating aget using cellulose nanofibers were developed applicable to the field of construction. As materials comprising the coating agent, cellulose nanofibers, AKD, waste glass powder (WGP), and Bisphenol A diglycidyl ether (BADGE) were used. Basic data were prepared for the development of coating agent technology in the field of construction using CNF.

## 2. Materials and Methods

### 2.1. Materials

CNF was purchased from Advanced Natural Polymer Inc. (Pohang, Republic of Korea). The CNF used is a TEMPO-oxidized cellulose nanofiber with fiber in water to a consistency of 2 wt%. The nominal properties of the CNF, as provided by the manufacturer, were fiber width 2–9 nm, conductivity 1.294 mS/cm, carboxylate contents 1.7 mmol/g, and crystallinity 61%. AKD was provided by Taewang Chemicals Co., Ltd. (Seoul, Republic of Korea). The nominal properties of AKD, as provided by the manufacturer, were solid content 20.2%, specific gravity 1.006, and viscosity 5.5 cps. WGP was collected in green glass bottles and washed under high pressure. After high-pressure washing, 1.0–10.0 μm of WGP was secured using a Micron-Master Jet Mill (Jet Pulverizer, Moorestown, NJ, USA). Bisphenol A diglycidyl ether was purchased from Sigma Aldrich (St. Louis, MO, USA).

### 2.2. Preparation of Coating Agent

BADGE was fixed at 0.7 of the total weight, and CNF, AKD, BADGE, and WGP were used as variables (Table 1). The cellulose nanofibers and distilled water were first stirred at 1000 rpm for 10 min, and then stirred at 3000 rpm for 30 min with a homogenizer while mixing AKD. BADGE was then incorporated at 1000 rpm for 30 min using a digital speed mechanical stirrer. The coating agent in which the waste glass fine powder was incorporated was stirred at 1000 rpm for 30 min before mixing with BADGE.

### 2.3. Coating Method

All coating materials were applied using a special coating brush. After stirring, they were immediately placed in a dry oven at 120 °C and heated for 24 h. The coating thickness was measured 10 times and the average value was used. The coating thickness was measured using a coating thickness meter (QNix 5500, Qnix, Bonn, Germany).

### 2.4. Test Method

#### 2.4.1. Fourier-Transform Infrared Spectroscopy (FTIR)

The Fourier-transform infrared spectroscopy (FTIR) spectra of materials and coating agent specimens (5 mg each) were acquired using an FTIR spectrometer (Spectrum 2, Perkin Elmer, Waltham, MA, USA). All samples were dried at 60 °C for 24 h before measurement.

#### 2.4.2. Contact Angle

ASTM D 5946 [50] was used to evaluate the contact angle of the coating agent. The water contact angles of the coating specimens were tested using a contact angle analyzer (DSA 100, KRUSS, Hamburg, Germany). A drop of 2 µL of deionized water was deposited on top of the coating specimens. The water contact angle was assessed 3 s after the drop of water was released on the surface, and the contact angle was measured three times, changing the surface. The contact angle test was conducted on cement mortar specimens, which had a thickness of 30 mm × 30 mm × 10 mm. Specimens with a 0.5 W/C ratio and 1:2 proportion of cement/sand were prepared. The specimens were vibrated for 2 min using a vibrating table machine, after which they were removed from the mold and then cured for 28 days. In a laboratory environment, the specimens were coated at a coating thickness of approximately 1000 μm.

#### 2.4.3. Hardness

Hardness measurements of all specimens were carried out using a pencil hardness tester (Wolff Wilborn, TQC, Zuid-Holland, The Netherlands), according to ASTM D 3363 [51]. The coated specimens were measured by placing them on a horizontal surface and holding a pencil to the durometer at an angle of 45° against the coating. A pencil with a hardness of 6H-6B was used for the test. Acrylic specimens with dimensions of 100 mm × 100 mm × 5 mm (thickness) were prepared.

#### 2.4.4. Rapid Chloride Ion Penetration Test (RCPT)

RCPT was conducted according to ASTM C 1202 [52] to evaluate the permeation resistance of the coating agent to chloride ions. Three identical concrete specimens with a diameter of 100 mm and thickness of 50 mm were prepared and coated on both sides. The concrete specimens were subjected to RCPT by applying 60 ± 1 V of direct current. The current was measured at 6 h. The chloride permeability was calculated using current and time in terms of coulombs. Coating agent was applied at a thickness of 1000 μm.

#### 2.4.5. Carbonation Test

Concrete specimens were subjected to accelerated carbonation in an environmental chamber at a temperature of 20 ± 2 °C and relative humidity of 65 ± 5%, and a CO_2_ concentration of 5.0 ± 0.2%. After carbonation was carried out via the accelerated carbonation test, the specimen was cut, and the carbonation penetration depth was measured using a color development method at the 28-day point using phenolphthalein solution.

#### 2.4.6. Marin Exposure Test

Structures exposed to the marine environment can generally be classified into splash zones, tidal zones, and underwater zones. Structures exposed to the underwater zone are partially affected by the chemical action of seawater, but corrosion of concrete rarely occurs due to the low supply of oxygen and carbon dioxide gas. Concrete exposed to the splash zone or tidal zone is usually in the worst state of deterioration of all the exposure categories; it is very important to regularly monitor this zone using a proper assessment method in order to reduce major repair and retrofitting work. 

In this study, the continuous performance of the coating agent in the marine environment was evaluated. The marine exposure test was conducted at Sihwa Lake in Ansan, Republic of Korea. The marine exposure test period was 6 months, and the test was conducted by applying the coating agent to concrete and steel specimens. The concrete specimen was manufactured to a size of 100 mm × 100 mm × 50 mm (thickness), and the steel test specimen was manufactured to a size of 100 mm × 100 mm × 4 mm (thickness). The marine exposure test specimens were set with OPC and with A10-W5-coated concrete and steel.

## 3. Results and Discussion

### 3.1. Fourier-Transform Infrared Spectroscopy (FTIR)

**Figure 1:**
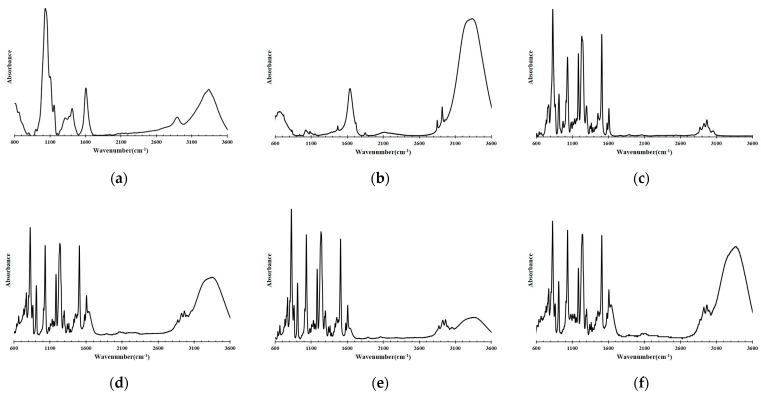
FTIR spectra representative of materials and coating agent specimens: (**a**) CNF; (**b**) AKD; (**c**) BADGE; (**d**) A10-W10; (**e**) A15-W5; (**f**) A10-W5-S.

Figure 1 shows the FTIR spectra when using the CNF, AKD, BADGE, and specimens of the coating agent. Figure 1a shows the FTIR spectra of CNF. The 3500 and 3200 cm^−1^ regions are due to the free O–H stretching vibration of hydroxyl groups in cellulose nanofibers [53]. The bands at around 2900 cm^−1^ relate to CH2 stretching vibrations. The regions of 1160 cm^−1^ and 1058 cm^−1^ are connected in turn to the C-O-C vibration and the C-O vibration connected to the hydroxyl group (addition). The peaks at 1420 cm^−1^ and 1610 cm^−1^ correspond to the C-O and C=O stretching of -COO^-^Na^+^ groups, respectively [54,55,56]. Figure 1b shows the FTIR spectra of AKD. The bands at 1131 cm^−1^ and 1086 cm^−1^ in the FTIR spectrum of the AKD correspond to the crystalline and amorphous phases, respectively [57]. The major spectral features of high-intensity peaks ranging from 2850 cm^−1^ and 2950 cm^−1^ are due to C-H stretching vibrations [58,59]. Figure 1c shows the FTIR spectra of BADGE. The BADGE spectrum shows typical aromatic bands at 1605 cm^−1^, 1508 cm^−1^, and 826 cm^−1^. The peaks at 1508 cm^−1^ and 827 cm^−1^ correspond to the C-H deformation vibration of BADGE [60,61]. Figure 1d–f show the FTIR spectra of the coating agent specimens. The spectrum of the coating agent shows an almost similar shape. The characteristic stretching vibrations at 845 cm^−1^ and 820 cm^−1^ and at 1350 cm^−1^ and 1275 cm^−1^ are associated with C-O-C stretching, in all specimens, indicating complete curing [62]. The broad bands around 3200 cm^−1^ and 3500 cm^−1^ correspond to the vibration mode of the OH group, which is released during the curing of the coating agent, which is observed in all specimens [63,64].

### 3.2. Contact Angle Test

**Figure 2:**
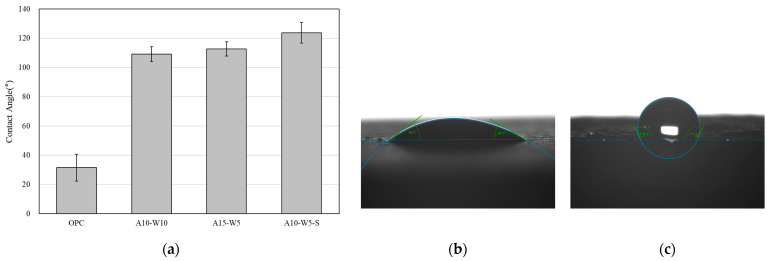
(**a**) Contact angle test result; (**b**) contact angle of OPC specimen; (**c**) contact angle of cement mortar with coating agent specimen.

Figure 2 shows the contact angle test results and the contact angle of the non-coated mortar specimens and coated specimens. The contact angle generally refers to wettability, which is the ability of a liquid to remain in contact with a surface. Depending on the size of the contact angle between the solid surface and water droplets, the material is classified into super-hydrophilic, hydrophilic, hydrophobic, and super-hydrophobic [65,66]. For the non-coated specimen, the contact angle was 31.55°. The contact angles of the cement mortar coated with the A10-W10 and A15-W5 coatings were 110.83° and 112.68°, respectively. The contact angle of A10-W5-S mixed with WGP was 123.65°. It is believed that WGP improved the hydrophobic evaluation by increasing the surface roughness.

### 3.3. Pencil Hardness

**Figure 3:**
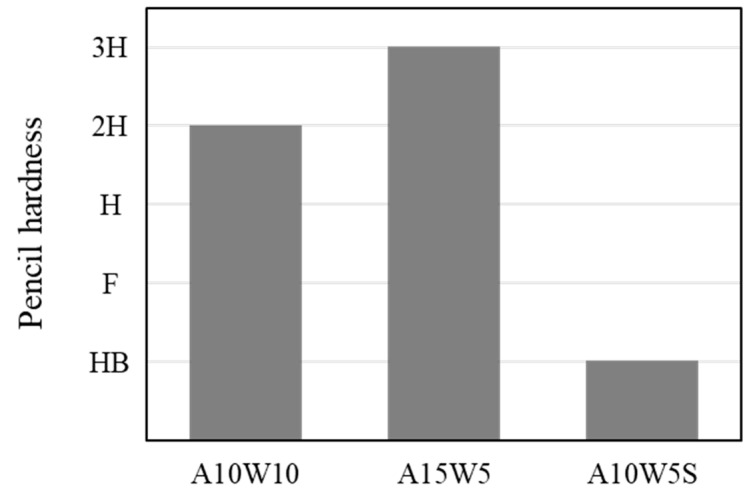
Hardness resistance of the coating agent.

The pencil scratching method was used to evaluate the hardness of the coating agent (Figure 3). The pencil hardness of A10-W10 was 2H. The pencil hardness of the A15-W5 coating agent with the high AKD content was 3H, one step higher. A10-W5-S mixed with WGP was confirmed to have a low HB pencil hardness due to an increase in surface roughness.

### 3.4. Rapid Chloride Ion Penetration Test (RCPT)

**Figure 4:**
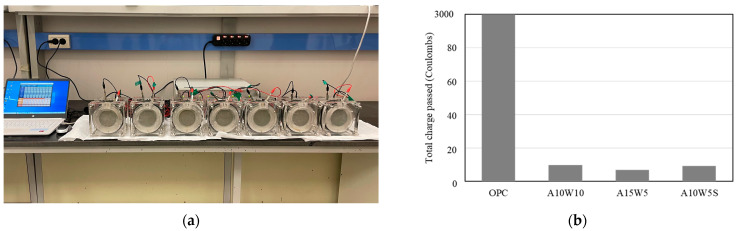
(**a**) Rapid chloride ion penetration test setup; (**b**) rapid chloride ion penetration resistance test results.

Figure 4 shows the chloride ion penetration resistance test results for the coating agent specimens. The RCPT values of the A10-W10, A15-W5, and A10-W5-S specimens decreased remarkably by 99.66%, 99.76%, and 99.70%, respectively, compared to the OPC specimens.

### 3.5. Carbonation Test

**Figure 5:**
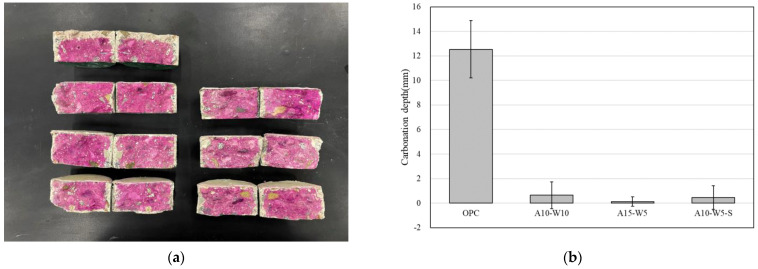
(**a**) The specimens after 28 days of exposure to the carbonation test; (**b**) carbonation test results.

Figure 5 compares the carbonation depths of the coated concrete specimens’ carbonation exposure test over 28 days. The test results reveal that the carbonation depth of the coated specimens is smaller than that of the non-coated specimens at the same age. The carbonation depths of the A10-W10, A15-W5, and A10-W5-S specimens decreased remarkably by 94.86%, 99.03%, and 96.38%, respectively, compared to the OPC specimens.

### 3.6. Marine Exposure Test

**Figure 6:**
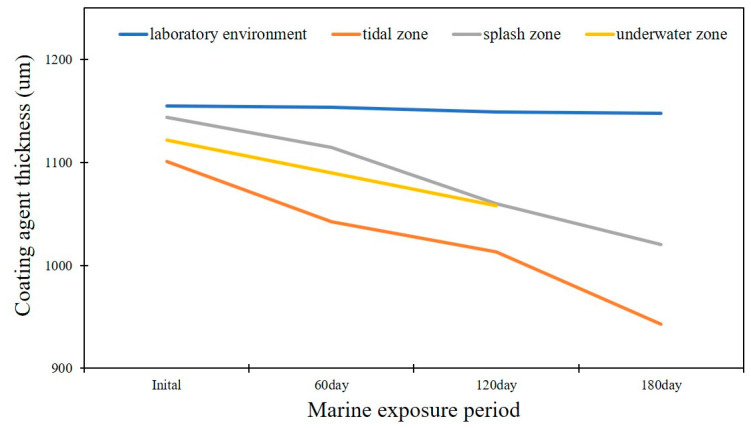
Coating thickness curve according to exposure environment and exposure period.

In the marine exposure test, the thickness of the coating agent was measured to evaluate the presence or absence of the coating. The coating thickness was measured before exposure to the marine environment; specimens were collected at intervals of about 60 days and the average coating thickness was repeatedly measured. The initial average coating thickness was over 1100 μm in all specimens. Figure 6 shows the measured coating thickness according to the exposure environment during the marine exposure period. In a laboratory environment, the coating thickness decreased by 7.2 μm for 180 days; however, the coating thickness of the specimens in the splash zone and tidal zone decreased by 123.5 μm and 158.4 μm, respectively. The specimens in the underwater environment were lost at 180 days. The coating thickness in the laboratory environment, tidal zone, splash zone, and underwater zone decreased by 0.52%, 7.79%, 7.35%, and 5.67%, respectively, compared to the initial thickness at 120 days of marine exposure. The coating thickness in the laboratory environment, tidal zone, and splash zone decreased by 0.62%, 14.39%, and 10.8%, respectively, compared to the initial thickness at 180 days of marine exposure.

**Figure 7:**
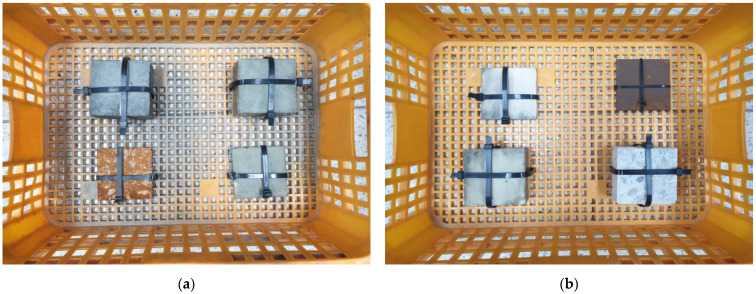
OPC and coated specimens exposed in the tidal zone and splash zone for 180 days. (**a**) Tidal zone; (**b**) splash zone.

Figure 7 shows the OPC and coated specimens exposed in the tidal zone and splash zone for 180 days. In the case of the splash zone marine environment, for the OPC concrete specimen, there was no significant difference in visual evaluation before and after the start of the marine environment exposure test. In contrast, in the case of the coating agent, relatively little wear was observed on some surfaces, and discoloration of the coating agent was also confirmed. In the case of the OPC steel test specimen, the color of the steel was confirmed to be dark brown, which is believed to be due to the stabilization of rust due to the occurrence of corrosion, and the corrosion rate gradually decreased. However, no corrosion occurred in the coated steel specimens.

In the case of the tidal zone marine environment, in the OPC concrete specimen, a significant difference before and after the test was confirmed with the naked eye compared to the early marine environment exposure test. On the surface of the concrete test specimen, large amounts of marine substances—such as seaweed and substances expected to be salt from seawater—were distributed throughout. It was confirmed that the coated concrete test specimen had fewer distributed substances expected to be salt compared to the OPC concrete specimen. In addition, it was confirmed that marine substances such as seaweed were similarly distributed. Unlike the steel test specimens exposed to the marine environment in the splash zone, the OPC steel test specimens were confirmed to have an orange color. The coated steel test specimen showed a similar appearance to the coated concrete specimen. In addition, corrosion of about 4.5 mm appeared at the point where the coating agent had not been properly applied.

## 4. Conclusions

The present experimental study was conducted on a hydrophobic coating agent developed using cellulose. In summary, hydrophobic coatings were developed using cellulose nanofiber, alkyl ketene dimer, waste glass powder, and Bisphenol A diglycidyl ether, and the maximum contact angle was 123.65°. A durability evaluation of concrete coated with the coating agent was performed. In the case of RCPT, a chlorine ion blocking effect of up to 99.76% was confirmed, and a resistance effect of carbonation penetration up to 99.03% was confirmed through the carbonation depth. The durability in the marine environment was confirmed through a marine exposure test for 6 months. Our approach can provide information for the development of eco-friendly coatings in the future.

## Figures and Tables

**Table 1:** Mix proportions of coating agent.

Specimen	CNF	AKD	Distilled Water	BADGE	WGP
A10-W10	0.1	0.1	0.1	0.7	-
A15-W5	0.1	0.15	0.05	0.7	-
A10-W5-S	0.1	0.1	0.05	0.7	0.05

## Data Availability

Not applicable.
